# Lights on Endocannabinoid-Mediated Synaptic Potentiation

**DOI:** 10.3389/fnmol.2020.00132

**Published:** 2020-07-28

**Authors:** Charlotte Piette, Yihui Cui, Nicolas Gervasi, Laurent Venance

**Affiliations:** ^1^Center for Interdisciplinary Research in Biology, College de France, INSERM U1050, CNRS UMR7241, Labex Memolife, Paris, France; ^2^Department of Neurobiology, Sir Run Run Shaw Hospital, Zhejiang University School of Medicine, Hangzhou, China

**Keywords:** endocannabinoids, synaptic plasticity, long-term potentiation, neuromodulation, GABAergic interneurons, cannabinoid receptor type-1, learning and memory, excitation-inhibition balance

## Abstract

The endocannabinoid (eCB) system is a lipid-based neurotransmitter complex that plays crucial roles in the neural control of learning and memory. The current model of eCB-mediated retrograde signaling is that eCBs released from postsynaptic elements travel retrogradely to presynaptic axon terminals, where they activate cannabinoid type-1 receptors (CB_1_Rs) and ultimately decrease neurotransmitter release on a short- or long-term scale. An increasing body of evidence has enlarged this view and shows that eCBs, besides depressing synaptic transmission, are also able to increase neurotransmitter release at multiple synapses of the brain. This indicates that eCBs act as bidirectional regulators of synaptic transmission and plasticity. Recently, studies unveiled links between the expression of eCB-mediated long-term potentiation (eCB-LTP) and learning, and between its dysregulation and several pathologies. In this review article, we first distinguish the various forms of eCB-LTP based on their mechanisms, resulting from homosynaptically or heterosynaptically-mediated processes. Next, we consider the neuromodulation of eCB-LTP, its behavioral impact on learning and memory, and finally, eCB-LTP disruptions in various pathologies and its potential as a therapeutic target in disorders such as stress coping, addiction, Alzheimer’s and Parkinson’s disease, and pain. Cannabis is gaining popularity as a recreational substance as well as a medicine, and multiple eCB-based drugs are under development. In this context, it is critical to understand eCB-mediated signaling in its multi-faceted complexity. Indeed, the bidirectional nature of eCB-based neuromodulation may offer an important key to interpret the functions of the eCB system and how it is impacted by cannabis and other drugs.

## Background

Endocannabinoids (eCBs) are a family molecule of biolipids, mainly composed by 2-arachidonoylglycerol (2-AG) and anandamide, synthesized and released on-demand, which mostly act on presynaptic cannabinoid type-1 receptors (CB_1_R) and postsynaptic transient receptor potential vanilloid type-1 (TRPV1; Piomelli et al., 2007; Castillo et al., 2012; Katona and Freund, 2012; Araque et al., 2017). eCBs have emerged as a major signaling system in learning and memory (Marsicano and Lafenêtre, 2009; Mechoulam and Parker, 2013; Kruk-Slomka et al., 2017) because of their powerful influence on synaptic plasticity, mainly as a depressing synaptic function (Castillo et al., 2012; Araque et al., 2017; Augustin and Lovinger, 2018). eCB signaling has been widely described to decrease the neurotransmitter release probability *via* diverse presynaptic mechanisms, including inhibition of voltage-gated calcium channels, activation of potassium channels, and protein kinase-A (pkA) signaling. In light of recent studies, this review aims at highlighting evidence for short and long-term eCB-mediated synaptic potentiation (eCB-LTP).

## eCB-Mediated Synaptic Potentiation

We have distinguished here the homosynaptic from heterosynaptic eCB-mediated potentiation such that homosynaptic plasticity refers to input-specific plasticity, in which only the neurons belonging to a given stimulated synapse undergo plasticity, whereas heterosynaptic plasticity refers to changes at a synapse resulting from activities of distinct synapses/pathways.

### Homosynaptic eCB-Mediated LTP

**Figure 1:**
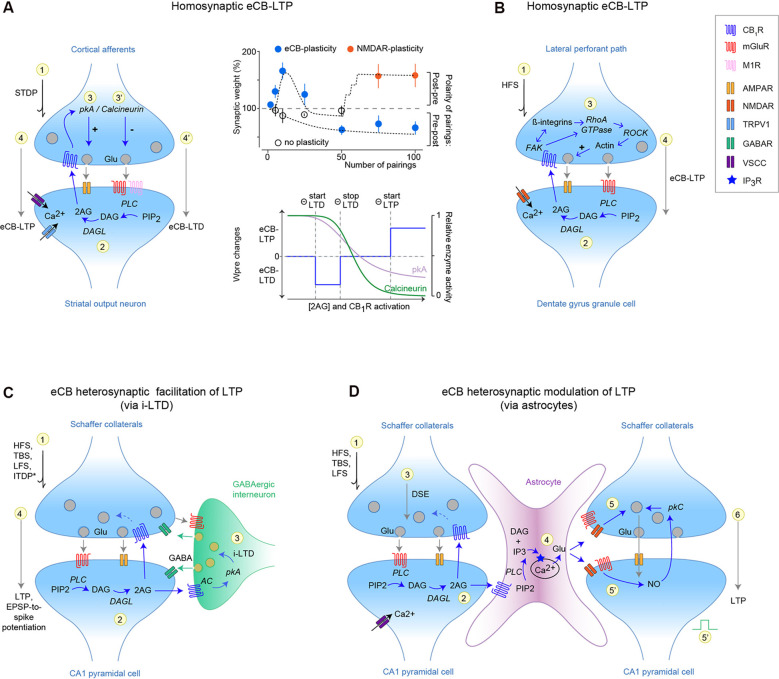
Homosynaptic and heterosynaptic endocannabinoid (eCB)-mediated long-term potentiation (LTP). **(A,B)** Homosynaptic eCB-LTP in the striatum **(A)** and hippocampus **(B)**. **(A)** eCB-LTP is induced by few spike-timing-dependent plasticity (STDP) pairings (~10–15 post-pre pairings), whereas eCB-LTD is induced by a larger number of pairings (~50–100 pre-post pairings) at the same corticostriatal synapses (Cui et al., 2015, 2016, 2018a; Xu et al., 2018; Gangarossa et al., 2020). Right: top panel illustrates the domains of expression of eCB-LTP, eCB-LTD, and NMDAR-LTP related to the polarity and number of STDP pairings. Bottom panel: eCB-LTP and eCB-LTD are expressed depending on eCB levels [and cannabinoid type-1 receptor (CB_1_R) activation], such that low and high levels induced eCB-LTD and eCB-LTP, respectively. CB_1_R activation is expected to decrease pkA and calcineurin activity, *via* reduced calcium influx through voltage-sensitive calcium channels (VSCC). This effect is schematized by the relative protein kinase-A (pkA) and calcineurin activity changes upon increased CB_1_R activation, such that eCB-LTD occurs when calcineurin/*pkA* >1, whereas calcineurin/*pkA* <1 leads to eCB-LTP. **(B)** eCB-LTP at synapses between the lateral perforant path (LPP) and the hippocampal granular cells of the dentate gyrus, requires co-operative CB_1_R/ROCK signaling, which favors glutamate release *via* a presynaptic actin regulatory mechanism (Wang et al., 2016, 2018b). eCB-LTP illustrated in **(A,B)** are independent of the GABAergic transmission and astrocytic calcium transients, present a presynaptic locus of expression and hence rely on a homosynaptic mechanism. **(C,D)** Heterosynaptic eCB-LTP involving intermediary elements such as GABAergic interneurons **(C)** and astrocytes **(D)**. **(C)** Heterosynaptic eCB-LTP at CA3–CA1 hippocampal synapses. Activation of mGluR1/5 promotes 2-AG synthesis and release; 2-AG activates CB_1_R located on neighboring GABAergic interneurons and induces LTD *via* a pkA-dependent mechanism. This LTD of inhibitory transmission (i-LTD) then facilitates the release of glutamate at CA3–CA1 synapses promoting a local LTP. This eCB-mediated metaplasticity can be induced with various cell conditioning paradigms, and most notably subthreshold stimulations (HFS, LFS, TBS or ITDP; Chevaleyre and Castillo, 2003, 2004; Zhu and Lovinger, 2007; Lin et al., 2011; Pan et al., 2011; Xu et al., 2012; Basu et al., 2013; Orr et al., 2014; Monory et al., 2015; Silva-Cruz et al., 2017; Kim et al., 2019). **(D)** Heterosynaptic eCB-LTP involving astrocytes at CA3–CA1 synapses. Neuronal activity (HFS, TBS, LFS) induces synthesis of eCBs at synapse-1 which activate astrocytic CB_1_R. Then, astrocytic glutamate release (*via* IP_3_-induced calcium-release mechanism) induces an NMDAR-facilitation or NO-mediated LTP on neighboring CA1 synapse-2. This eCB-mediated lateral synaptic regulation has been observed in the hippocampus (Navarrete and Araque, 2008, 2010; Gómez-Gonzalo et al., 2015; Covelo and Araque, 2016) and striatum (Martín et al., 2015).

Using spike-timing-dependent plasticity (STDP), a Hebbian synaptic learning rule relying on paired activity on either side of the synapses (Feldman, 2012), a few numbers of pairings induce eCB-LTP at corticostriatal synapses, which is CB_1_R- and TRPV1-mediated (Cui et al., 2015, 2016, 2018a; Xu et al., 2018; Figure 1A). 2-AG levels and subsequent CB_1_R activation have a dual effect on eCB-plasticity: high levels of eCBs synthesis and CB_1_R activation (reached with ~10–15 post-pre pairings) induce eCB-LTP, while low levels (reached with ~50–100 pre-post pairings) induce eCB-LTD (Cui et al., 2015, 2016). Indeed, few pairings promote efficient eCB synthesis (*via* maximal calcium influx and efflux from voltage-gated calcium channels and TRPV1, and endoplasmic reticulum, respectively) and thus maximal CB_1_R activation, combined with minimal CB_1_R desensitization (Cui et al., 2016). Corticostriatal eCB-plasticity relies on presynaptic pkA/calcineurin balance, such that eCB-LTP requires active pkA, whereas eCB-LTD depends on calcineurin activation (Cui et al., 2016; Figure 1A). Therefore, at corticostriatal synapses, eCB-mediated plasticity is bidirectional, and eCB-LTP or eCB-LTD expression is determined by pre- and postsynaptic activity patterns. A similar form of homosynaptic and bidirectional eCB-plasticity occurs between neocortical pyramidal cells following a limited number of coincident activity (Cui et al., 2018b). Interestingly, eCB-LTP is robust to spike-time jittering, contrarily to NMDAR-LTP, and can thus arise in noisy neural network activity (Cui et al., 2018a).

A homosynaptic CB_1_R-dependent eCB-LTP was also characterized in hippocampal granular cells of the dentate gyrus resulting from postsynaptic 2-AG synthesis upon high-frequency stimulation of the lateral perforant path (LPP; Wang et al., 2016, 2018a; Figure 1B). When activated, CB_1_R, detected presynaptically at LPP terminals using STORM microscopy, engage the presynaptic FAK/ROCK signaling pathway favoring glutamate release. Interestingly, at CA3-CA1 synapses CB_1_R is preferentially linked to ERK/Munc18–1, whose activation depresses glutamate release (Wang et al., 2018a).

In both cases, the eCB-LTP magnitude did not reach saturating levels and could be increased under monoacylglycerol lipase (MAGL) inhibition, the 2-AG degrading enzyme, suggesting that eCB-LTP might serve as a priming plasticity accounting for fast learning and episodic memory. Finally, homosynaptic CB_1_R-mediated eCB-LTP was also observed in stratum oriens interneurons (Friend et al., 2019).

### eCB-Mediated Heterosynaptic Facilitation of LTP

#### *Via* Depression of Inhibitory Transmission

By reducing inhibition from GABAergic synapses through a CB_1_R-dependent short-term depolarization-induced suppression of inhibition (DSI), eCBs were first shown to facilitate NMDAR-LTP induction at hippocampal CA3-CA1 synapses (Carlson et al., 2002), exclusively in the cell subjected to the subthreshold LTP inducing protocol. eCB-mediated facilitation through long-term disinhibition was then observed at various synapses, cell types, and brain regions. Indeed, in the hippocampus, high or low-frequency stimulations or theta-burst stimulations of Schaffer collaterals induce LTD of local GABAergic interneurons (i-LTD), which in turn facilitates LTP at excitatory CA3-CA1 synapses (Chevaleyre and Castillo, 2003, 2004; Zhu and Lovinger, 2007; Lin et al., 2011; Pan et al., 2011; Xu et al., 2012; Monory et al., 2015; Silva-Cruz et al., 2017; Figure 1C). i-LTD, originating from metabotropic glutamatergic receptor (mGluR) activation and subsequent 2-AG release from CA1 pyramidal cells that leads to the activation of CB_1_R located on GABAergic terminals, causes relief of the GABAergic brake in a restricted dendritic area (~10 μm) when synaptically-induced or on a cell-wide extent following endogenous CA1 pyramidal cell activity (Younts et al., 2013). In contrast to the transient LTP facilitation induced by DSI in single active cell and up to neighboring naïve cells (Wilson and Nicoll, 2001), i-LTD provides long-lasting priming of at most a single cell (Chevaleyre and Castillo, 2004; Younts et al., 2013). The modulation of CA1-LTP by i-LTD is an example of metaplasticity, *i.e*. long-lasting neural changes induced by activity at a given time, and that modulate subsequently induced plasticity (Abraham, 2008), orchestrated by eCBs. This i-LTD is finely tuned by the parallel activation of CB_1_R on GABAergic or glutamatergic cells (Monory et al., 2015) and is also accompanied by changes in excitability enhancing the spiking probability in response to a given EPSP, *i.e*., EPSP-to-spike potentiation (Chevaleyre and Castillo, 2003; Orr et al., 2014; Kim et al., 2019), and by structural changes (Monory et al., 2015; Hu et al., 2019) both eCB-mediated. Interestingly, a circuit-based synaptic learning rule, consisting of paired stimulation of the perforant path and Schaffer collaterals, induced an input-timing-dependent heterosynaptic LTP at CA3-CA1 but not at cortical-CA1 synapses (Xu et al., 2012; Basu et al., 2013). Input-timing-dependent-LTP depends on CB_1_R-mediated i-LTD occurring at GABAergic synapses (here cholecystokinin interneurons). Activation of the cortical-CA1 pathway triggers heterosynaptic calcium transients, boosting eCB signaling originating from the CA3-CA1 pathway, which leads ultimately to i-LTD. Similar metaplasticities involving eCB-mediated i-LTD have been reported in the striatum (Adermark, 2011; Mathur et al., 2013), ventral tegmental area (Szabo et al., 2002), basolateral amygdala (BLA; Azad et al., 2004) and spinal cord (Kyriakatos and El Manira, 2007).

#### *Via* Astrocytes

eCBs, released from a given stimulated CA3–CA1 synapse, activate astrocytic CB_1_R and *via* an IP_3_-induced calcium-release mechanism promote astrocytic glutamate release, which in turn induces an NMDAR-mediated short- (Navarrete and Araque, 2008, 2010) or nitric oxide(NO)-mediated long-term (Gómez-Gonzalo et al., 2015) potentiation on neighboring CA1 synapses (Figure 1B). This lateral synaptic regulation achieved by astrocytes and eCBs (Covelo and Araque, 2016), also reported in the dorsal striatum (Martín et al., 2015), appears as a means of controlling distant synapses by activated ones. Since, astrocytes are interconnected by gap junctions, permeable to calcium and IP_3_, both involved in the propagation of intercellular calcium waves (Giaume and Venance, 1998), the role of astrocytic gap junctions in regulating the extent of this lateral synaptic regulation remains to be determined.

#### *Via* Dopaminergic Signaling

At the goldfish Mauthner cell, sustained activity at excitatory synapses triggers 2-AG release, which activates CB_1_R on nearby dopaminergic fibers and promotes an increased release of dopamine (Cachope et al., 2007). In turn, dopamine acts back *via* a D_1/5_R-mediated pkA signaling, which induces LTP at electrical and glutamatergic chemical synapses.

### Non-CB_1_R-Mediated eCB-Potentiation of Synaptic Transmission

In the hippocampus, anandamide induces an increase of miniature excitatory (Sang et al., 2010) and inhibitory (Hofmann et al., 2011) postsynaptic currents. Anandamide and 2-AG potentiate NMDAR-mediated currents *via* respectively TRPV1-dependent and -independent mechanisms (Hampson et al., 1998; Yang et al., 2014). Although, this latter anandamide/2-AG NMDAR-mediated metaplasticity favors hippocampal LTD (Yang et al., 2014), it remains to investigate whether this eCB-NMDAR cross-talk exists in other brain areas and, considering the crucial role of NMDAR in synaptic potentiation, could constitute a metaplasticity promoting LTP.

## Neuromodulation of eCB-LTP

eCB-LTP expression or magnitude can be regulated by neuromodulators through a variety of mechanisms targeting eCB synthesis and/or release, or the signaling downstream of CB_1_R.

### Dopamine

The relationship between dopamine and eCB-signaling has been extensively documented for eCB-LTD (Covey et al., 2017). Recent evidence also shows a tight link between dopamine and eCB-LTP. In the globus pallidus, eCB-mediated i-LTD is switched to i-LTP upon D_2_R activation (Caballero-Florán et al., 2016). Striatal homosynaptic eCB-LTP is prevented when STDP pairings are applied simultaneously to opto-inhibition of nigrostriatal dopaminergic neurons and depends on presynaptic D_2_R located on cortical afferents, whose activity level shapes the expression domain of eCB-LTP and eCB-LTD (Xu et al., 2018). Interestingly, restricting Gi/o protein availability in presynaptic terminals switches the coupling of CB_1_R to Gs and stimulates pkA pathway (Glass and Felder, 1997; Gonzalez et al., 2009): this competition for Gi/o availability between CB_1_R and D_2_R could favor presynaptic pkA activation and thus promote corticostriatal eCB-LTP (Cui et al., 2016).

### GABA

GABA acts as a Hebbian/anti-Hebbian switch, which orientates the polarity of corticostriatal homosynaptic eCB-LTP: eCB-LTP is induced by post-pre pairings in native conditions, but by pre-post pairings under GABAergic transmission blockade (Cui et al., 2015).

### NO

Biological actions of eCBs partly rely on their ability to regulate NO signaling (Lipina and Hundal, 2017). At cerebellar parallel fiber-Purkinje cell synapses, low and high-frequency stimulations induce differential CB_1_R activation leading to low and high amount of NO production, which orientates the plasticity, respectively, towards eCB-LTP and eCB-LTD (Wang et al., 2014). Therefore, NO levels may act as a threshold in the modulation of synaptic strength (Song et al., 2012; Wang et al., 2014).

### Brain-Derived Neurotrophic Factor (BDNF)

BDNF modulates not only eCB-LTD (Heifets and Castillo, 2009) but also eCB-LTP. For heterosynaptic eCB-LTP in the hippocampus, neocortex, ventral tegmental area, and striatum, activation of the postsynaptic tropomyosin receptor kinase-B (TrkB) by BDNF increases 2-AG mobilization and consequently CB_1_R activation, which allows an eCB-mediated depression of IPSCs (Lemtiri-Chlieh and Levine, 2010; Selvam et al., 2018) and i-LTD (Zhao et al., 2015; Zhong et al., 2015), tuning the magnitude of glutamatergic LTP. In the neocortex, eCBs released by dendritic calcium spikes reduce inhibitory transmission, which facilitates postsynaptic calcium spike generation, the calcium-dependent release of BDNF, and ultimately the induction of eCB-LTP (Maglio et al., 2018). For homosynaptic eCB-LTP, TrkB activation facilitates 2-AG synthesis and shapes the expression domain of corticostriatal eCB-LTP (Gangarossa et al., 2020).

## eCB-Mediated LTP in Learning

While several links between eCB-LTD and various forms of memories have been woven, such as in habit learning or during critical periods of sensory processing (Augustin and Lovinger, 2018), we focus here on the recent starting evidence of the involvement of homosynaptic eCB-LTP and eCB-mediated heterosynaptic facilitation of LTP in learning, based on studies using electrophysiological recordings, and pharmacological or genetic tools modifying eCB-LTP.

### Homosynaptic eCB-LTP

At the LPP-dentate gyrus synapses, conveying cue identity to the hippocampus, eCB-LTP is implicated in memory of both simultaneous and serial two-odor discriminations, acquired after a small number of trials in rats (Wang et al., 2016, 2018a,b). Systemic injections of CB_1_R or MAGL antagonist, preventing or enhancing, respectively, eCB-LTP, had opposite effects on learning of the simultaneous two-odor discrimination task. Importantly, MAGL inhibition led to long-term memory 24 h after six training trials, a protocol which failed to induce efficient learning in controls (Wang et al., 2016). Moreover, learning performance was correlated with greater expression of pROCK in LPP of trained rats and reduced expression with CB_1_R inhibitor (Wang et al., 2018b). The serial odor discrimination task, testing the encoding of cues embedded in a sequence, is thought to reflect the constant flow of experience characteristic of episodic memory (Wang et al., 2018a). *Frm1*-KO mice, characterized by impaired 2-AG signaling, reduced NMDAR-mediated transmission, and a strong impairment of eCB-LTP at LPP-dentate gyrus synapses, show learning deficits in this task. Also, systemic injection of MAGL inhibitor or chemogenetic activation of Gq in the entorhinal cortex was sufficient to rescue *in vitro* homosynaptic eCB-LTP and learning.

### Heterosynaptic eCB-Mediated Facilitation of LTP

**Figure 2:**
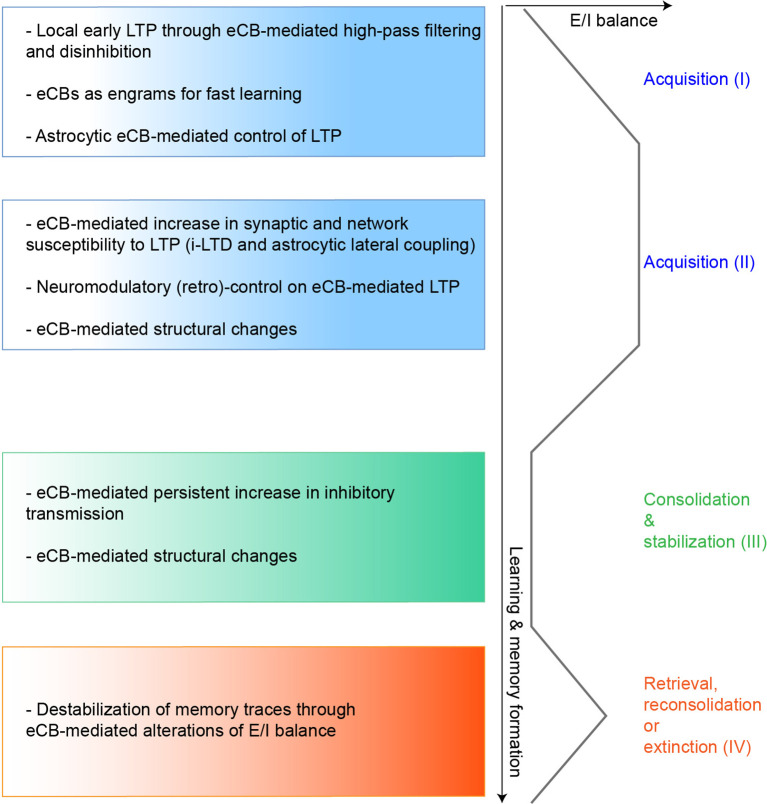
Hypothetical model of eCBs functions in regulating LTP during learning. We propose here a speculative model of eCBs contribution in controlling LTP expression and the excitation/inhibition (E/I) balance during the different learning stages, based on the mechanisms described in several *in vitro* and *in vivo* studies. During the initial phases of memory acquisition (I) eCBs could initially operate as high-pass filters, favoring LTP at strongly active synapses (Silva-Cruz et al., 2017). Also, eCBs-mediated disinhibitory mechanisms (such as DSI or i-LTD operating at different scales) could induce LTP at specific excitatory synapses and fine-tune the E/I balance during learning (Chevaleyre and Castillo, 2003; Xu et al., 2014; Busquets-Garcia et al., 2018). In parallel, eCBs, whose main modus operandi is on-demand biosynthesis and release, behave as highly sensitive and robust detectors of synaptic activity, allowing LTP induction even after a few jittered coincident activity patterns (Cui et al., 2015, 2018a). This feature may thus be used during fast learning and could contribute to episodic memory (Wang et al., 2016). Finally, eCBs can control the astrocytic-dependent release of co-factors necessary for LTP induction (Robin et al., 2018). In the later phases of memory acquisition (II), eCBs could increase the network susceptibility to synaptic modifications, near LTP induction focal points, with more or less spatial extent through heterosynaptic plasticity mechanisms (Chevaleyre and Castillo, 2003; Gómez-Gonzalo et al., 2015; Martín et al., 2015). Importantly, since eCBs can act as bimodal regulators of synaptic plasticity and interact with several neuromodulators, eCB-mediated LTP could also be switched back to normal, or turned to depression (Wang et al., 2014; Caballero-Florán et al., 2016; Xu et al., 2018), depending for instance on late behavioral outcomes, such as in reinforcement learning. During consolidation (III) eCB-mediated potentiation of inhibitory transmission and structural synaptic changes can stabilize acquired memory engrams (Monory et al., 2015; Ghosh et al., 2018; Hu et al., 2019). Finally, reactivation of memories (IV) especially emotional ones, increases eCB signaling, which can operate in feedback and control the lability of these memory traces by modifying the E/I balance (Li et al., 2008; Segev et al., 2018).

Several studies highlight the importance of eCB-mediated i-LTD and the regulation by eCBs of the excitation/inhibition balance in memory formation and maintenance (Figure 2), unraveling a novel role of eCBs in disinhibitory mechanisms during learning (Letzkus et al., 2015). Mice in which only sub-saturating forms of LTP requiring i-LTD expression *in vitro* were impaired in CA1 pyramidal cells (by a targeted mGluR5 knock-out), showed no deficits in spatial memory but performed poorly in trace-conditioning tasks when a long 30 s interval separated the two cues (Xu et al., 2014). Although, partial occlusion of i-LTD in wild-type mice could be observed *ex vivo*, LTP could still be induced in CA1 after learning, probably because only a few active synapses had been saturated during the task. The acquisition and retention of this temporally-based associative learning were enhanced by systematic MAGL inhibition, shown to promote i-LTD-mediated LTP. This echoed a previous study (Pan et al., 2011), in which MAGL knock-out mice showed improved learning in the water maze and object recognition tasks (but see Griebel et al., 2015). In the same vein, a subpopulation of hippocampal interneurons appears critical in controlling the level of inhibition and CB_1_R- dependent LTP expression in pyramidal neurons during incidental learning: GABA-CB_1_R-KO mice have impaired learning and *in vivo* LTP, which can both be fully rescued by reducing GABAergic transmission. Furthermore, enhancement of *ex vivo* i-LTD amplitude in trained mice suggests its involvement in learning (Busquets-Garcia et al., 2018). CB_1_R activation is also required for encoding emotionally salient stimuli at the BLA-medial prefrontal cortex pathway (Laviolette and Grace, 2006; Tan et al., 2010, 2011): notably BLA pharmacological CB_1_R activation or anandamide reuptake inhibitor potentiates the formation of associative memories with normally subthreshold footshock, putatively through CB_1_R-dependent heterosynaptic facilitation of BLA output (Azad et al., 2004), and leads to enhanced cortical activity and bursting in response to olfactory cues previously paired with footshock. Systemic treatment with CB_1_R antagonist prevents *in vivo* LTP expression and learning. Finally, astroglial CB_1_R-knock-out mice do not express NMDAR-LTP at CA3–CA1 synapses *in vivo* and show impaired performance in the novel object recognition task (Robin et al., 2018), which could be rescued by elevating D-serine levels, gating NMDAR activation.

Interestingly, a long-lasting enhancement of inhibitory transmission observed in cortical neurons *ex vivo* after training on a difficult olfactory discrimination task relies on an unusual eCB-mediated mechanism: post-synaptic persistent CB_1_R activation in pyramidal cells leads to an inhibition of pkA, which induces an increase in postsynaptic GABA_A_ channel conductance (Ghosh et al., 2018).

Overall, the learning of several tasks might initially be associated with an eCB-mediated relief of GABAergic transmission, hence reducing the threshold of LTP induction and could be followed by an elevation of the inhibitory tone that could participate to long-term memory stabilization (Figure 2).

## Therapeutic Perspectives

eCBs have long been involved in several brain disorders, in particular drug addiction and pain (Araque et al., 2017). Most of the dysregulation of eCB-mediated LTP described below involve heterosynaptic facilitation of LTP through disinhibitory mechanisms.

### Stress Coping

Acute and chronic stress reduce anandamide levels and modify 2-AG signaling and CB_1_R expression (Ruehle et al., 2012; Morena et al., 2016), and lead to persistent changes in eCB-mediated plasticity expression and polarity *ex vivo* (Glangetas et al., 2013; Bosch-Bouju et al., 2016) and *in vivo* (Segev et al., 2018). Mimicking anandamide reduction by selectively overexpressing FAAH at hippocampal CA3-CA1 synapses led to increased anxiety along with an enhancement of LTP expression *in vitro*, while i-LTD and DSI remained unchanged (Zimmermann et al., 2019). Yet, FAAH overexpression in BLA pyramidal neurons can also attenuate stress and anxiety-like behaviors (Morena et al., 2019): as an explanation, FAAH overexpression could dry out tonic anandamide signaling at GABAergic synapses and shift the excitation/inhibition balance towards inhibition of BLA output neurons. These results highlight the need for considering the excitatory/inhibitory nature of neurons where CB_1_R is activated to understand the impact of plasticity changes at the network output level.

Conversely, evidence for elevated anandamide during extinction training corroborates with the persistent facilitation of fear extinction induced by pharmacologically increasing anandamide levels in BLA, hippocampus or mPFC (Lin et al., 2008; Gunduz-Cinar et al., 2012; Shoshan et al., 2017; Segev et al., 2018) and with its impairment by CB_1_R antagonists or FAAH overexpression (De Oliveira Alvares et al., 2008; Lin et al., 2008; Abush and Akirav, 2009; Gunduz-Cinar et al., 2012; Zimmermann et al., 2019). In particular, while increased FAAH activity is observed in BLA and hippocampus following shock exposure, local application of FAAH inhibitor renormalizes stress-induced plasticity changes, re-allowing CA1-CA3 LTP expression while causing a decrease of BLA LTP *in vivo* (Segev et al., 2018). These manipulations operated immediately or 24 h after a situational reminder of fear-conditioning, persistently attenuated fear expression in mice. Yet, enhanced BLA i-LTD was also reported after local administration of FAAH inhibitor under stressed conditions, and could selectively enhance neuronal excitability in specific BLA glutamatergic networks (Azad et al., 2004; Gunduz-Cinar et al., 2012). Activation of hippocampal TRPV1, which was shown to enhance CA1 LTP *via* the GABAergic system *in vitro* (Bennion et al., 2011), could prevent the stress-induced switch from LTP to LTD and stress-induced impairment of spatial memory retrieval (Li et al., 2008). Overall, a targeted elevation of eCBs appears as a strategy for coping with stress, with preliminary clinical applications (Papagianni and Stevenson, 2019; Mayo et al., 2020).

### Drug Addiction

The facilitation of LTP in dopaminergic neurons is reported after exposure to cocaine, ethanol, or nicotine (Parsons and Hurd, 2015). For prolonged cocaine exposure, such facilitation, accompanied by increased bursting of dopaminergic neurons, is likely mediated by an eCB-dependent disinhibitory feedback loop (Liu et al., 2005; Pan et al., 2008a,b). Indeed, cocaine intake occludes *ex vivo* eCB-mediated i-LTD while manipulating eCBs signaling (by local application of CB1R or mGluR5 antagonists, or by blocking 2-AG synthesis) alleviates cocaine-induced reduction of inhibitory transmission (Pan et al., 2008b; Wang et al., 2015; Zhong et al., 2015). Chronic nicotine self-administration facilitates the induction of CB_1_R-mediated LTP in the bed nucleus of the stria terminalis, and this facilitation resists to a long period of forced abstinence (Reisiger et al., 2014). As this area is involved in cue-induced drug-seeking, these persistent changes could be responsible for increased vulnerability to relapse.

### Alzheimer and Parkinson’s Diseases

In Alzheimer’s disease, β-amyloid accumulation results in a reduction of hippocampal LTP, and notably prevents eCB-mediated disinhibition and EPSP-to-spike potentiation *in vitro* (Orr et al., 2014), a phenomenon that could contribute to memory deficits. Knocking-out CB_1_R in an Alzheimer mouse model worsens learning impairments, while treatments with an eCB-reuptake inhibitor or exogenous cannabinoids improve memory (Bedse et al., 2014). In parkinsonian rodents, striatal homosynaptic eCB-LTP is prevented *ex vivo* and can be rescued by Levodopa (Xu et al., 2018), and the globus pallidus exhibits a reduced GABAergic transmission, which is reversed by the co-activation of D_2_R and CB_1_R (Muñoz-Arenas et al., 2015).

### Pain

In the spinal cord, eCBs have both anti- and pro-nociceptive effects through inversed plasticity mechanisms, respectively by depressing nociceptive and disinhibiting non-nociceptive afferents (Pernia-Andrade et al., 2009; Kato et al., 2012). The underlying mechanism was evidenced in the semi-intact preparation of the nervous ganglia of the medicinal leech, in which eCB-mediated heterosynaptic potentiation of non-nociceptive synapses is critical to producing behavioral sensitization in response to noxious stimuli (Higgins et al., 2013; Wang and Burrell, 2018).

## Conclusions

Although, it has encountered some skepticism at times, various forms of eCB-mediated LTP have been characterized in different brain areas. If their involvement in memory has been proposed by a substantial body of experimental evidence, further work is necessary to make a direct link between the two in several paradigms, using targeted *in vivo* recordings and pharmacological or genetic manipulations. eCB-LTP should not be viewed as an unconventional or atypical form of eCB-plasticity, but as the other side of the eCB-mediated engram, making eCBs bidirectional regulators of synaptic plasticity, similarly to most neurotransmitters. With the increasing promising applications for cannabis and eCB-based drugs in medicine, we need to consider eCBs bidirectional effects, which also expand considerably their potential field of therapeutic applications.

## Author Contributions

CP wrote the “eCB-Mediated LTP in Learning,” “Therapeutic Perspectives,” and “eCB-Mediated Synaptic Potentiation” sections, and designed the Figures 1, 2. YC wrote the “eCB-Mediated Synaptic Potentiation” section. NG wrote the “Neuromodulation of eCB-LTP” section. LV wrote the “eCB-Mediated Synaptic Potentiation,” “Non-CB1R-Mediated eCB-Potentiation of Synaptic Transmission,” and “Conclusions” sections, and designed the Figure 1. All authors have edited and corrected the manuscript.

## Conflict of Interest

The authors declare that the research was conducted in the absence of any commercial or financial relationships that could be construed as a potential conflict of interest.

## Abbreviations

2-AG: 2-arachidonoylglycerol
AMPAR: α-amino-3-hydroxy-5-methyl-4-isoxazolepropionic acid receptor
BDNF: brain-derived neurotrophic factor
BLA: basolateral amygdala
CB_1_R: cannabinoid type-1 receptor
DSI: depolarization-induced suppression of inhibition
DXR: dopaminergic type-X receptor
E/I balance: excitation/inhibition balance
FAAH: fatty acid amide hydrolase
eCB: endocannabinoid
GABA: gamma-aminobutyric acid
i-LTD: long-term depression of inhibitory transmission
LPP: lateral perforant path
LTP: long-term potentiation
LTD: long-term depression
MAGL: monoacylglycerol lipase
mGluR: metabotropic glutamatergic receptor
mPFC: medial prefrontal cortex
NMDAR: N-methyl-D-aspartate receptor
NO: nitric oxide
pkA: protein kinase-A
STDP: spike-timing-dependent plasticity
TrkB: tropomyosin receptor kinase-B
TRPV1: transient receptor potential vanilloid type-1.
